# Effects of Hyponatremia Normalization on the Short-Term Mortality and Rehospitalizations in Patients with Recent Acute Decompensated Heart Failure: A Retrospective Study

**DOI:** 10.3390/jcm5100092

**Published:** 2016-10-22

**Authors:** Renato De Vecchis, Marco Di Maio, Giuseppina Di Biase, Carmelina Ariano

**Affiliations:** 1 Cardiology Unit, Presidio Sanitario Intermedio ”Elena d’Aosta”, ASL Napoli 1 Centro, via Cagnazzi 29, 80137 Napoli, Italy; 2 Department of Cardiology, Second University of Napoli, Monaldi Hospital, via Leonardo Bianchi 1, 80131 Napoli, Italy; marcodimaio88@gmail.com; 3 Division of Geriatrics, Neurorehabilitation Unit, Clinic “S. Maria del Pozzo”, via Pomigliano 40, 80049 Somma Vesuviana, Italy; smariaves268@gmail.com; 4 Division of Cardiology, Casa di Cura “Sollievo della Sofferenza”, viale Cappuccini 2, 71013 San Giovanni Rotondo, Italy; carmelariano@tiscali.it

**Keywords:** heart failure, hyponatremia, mortality, hospitalization

## Abstract

**Background:** Several studies have shown that hyponatremia is associated with increased risk of rehospitalization and death in patients with heart failure. In these studies, chronic heart failure (CHF) patients with persistent hyponatremia were compared only with CHF patients with a normal sodium level at hospital admission. **Aims:** In the present retrospective study, conducted in a cohort of patients with recent acute decompensated heart failure (ADHF), all with hyponatremia ascertained at the time of hospital admission, we aimed to evaluate the effect of the normalization of serum sodium on the composite endpoint of short-term rehospitalization and mortality. **Methods:** A retrospective study centered on medical records of patients hospitalized for ADHF in the period April 2013 to April 2016 was performed. Data regarding serum sodium measurements had to be collected from medical records of cardiology wards of two hospitals, and were then processed for statistical analysis. As an inclusion criterion for enrollment, patients had to be suffering from heart failure that had required at least one hospitalization. Moreover, they had to be suffering from a state of hyponatremia (serum sodium < 135 mEq/L) at admission on the occasion of the index hospitalization. Patients with hyponatremia at admission were divided into two groups, one comprising patients with hyponatremia that persisted at the time of discharge (persistent hyponatremia) and a second including patients who had achieved normalization of their serum sodium levels (serum Na^+^ ≥ 135 mEq/L) during hospitalization until discharge. For both groups, the risk of mortality and rehospitalization during a 30-day follow-up was assessed. **Results:** One hundred and sixty CHF patients with various degrees of functional impairment were enrolled in the study. Among them, 56 (35%) had persistent hyponatremia over the course of hospitalization. At multivariable Cox proportional-hazards regression analysis, the risk of having a 30-day unplanned readmission or death was significantly higher in patients with persistent hyponatremia compared to those who exhibited a sodium level normalized at discharge (adjusted hazard ratio = 3.0743; 95% CI: 1.3981–6.7601; *p* = 0.0054). Among the other variables included in the Cox regression model, the number of admissions in the last 12 months (*p* < 0.0001), the length of stay of the index admission (*p* = 0.0015) and the New York Heart Association (NYHA) class III at discharge (*p* = 0.0022) were also identified as risk factors associated with the composite endpoint of 30-day unplanned readmission or death. **Conclusions:** In the present retrospective study, the risk of 30-day rehospitalization or death was significantly higher in patients with recent ADHF and persistent hyponatremia in comparison with ADHF patients who had had their serum sodium normalized during the hospital stay. This association seemed to be independent of the heart failure severity.

## 1. Background

Several studies have shown that hyponatremia is associated with increased risk of rehospitalization and death in patients with heart failure [1,2,3,4,5]. In these studies, patients with chronic heart failure (CHF) and persistent hyponatremia were compared only with CHF patients with a normal sodium level at hospital admission. By contrast, in our study we decided to retrospectively study a cohort of patients hospitalized for acute decompensated heart failure (ADHF), all characterized by the finding of hyponatremia at admission. Indeed, in the case that normalization of hyponatremia during hospitalization was achieved, thanks to general therapeutic measures (optimization of drug therapy for heart failure) or specific therapeutic protocols (restriction of dietary water intake, administration of combination therapy with furosemide and hypertonic saline, use of tolvaptan in association with loop diuretics), we assumed that clinical outcomes of mortality and rehospitalization in the short term, i.e., at a follow-up of 30 days, might have been improved accordingly. Our working hypothesis was not really supported by existing literature data. Indeed, studies that evaluated the impact of hyponatremia on hard clinical endpoints (cardiovascular mortality, all-cause mortality, rehospitalization due to acute exacerbation of heart failure) have considered patients with heart failure and hyponatremia persistent at discharge by comparing them with heart failure patients who had normal serum sodium values at the time of admission. In addition, studies that addressed the issue of the possible favorable impact on short-term clinical outcomes achievable by means of a normalization of the hyponatremia during hospitalization provided conflicting results [6,7,8].

## 2. Methods

A retrospective study was conducted by examining medical records of patients with a history of ADHF and hyponatremia at admission dating back to the period April 2013 to April 2016. The case records were obtained from the archives of medical records in paper and/or electronic format of two hospitals (Casa di Cura “Sollievo della Sofferenza”, S. Giovanni Rotondo, Italy, and Clinica S. Maria del Pozzo, Somma Vesuviana, Italy). In the course of data collection, close attention was paid to comply with the current rules and regulations aimed to preserve the anonymity and privacy of recruited patients. Data were then processed in a third center (Presidio Sanitario Intermedio “Elena d’Aosta”, Napoli, Italy) with their incorporation into a multivariable Cox proportional-hazards regression model. Data collection and statistical analysis were performed after consent of the Health Directorates of the hospitals involved in the research. Patients were enrolled in the study if the two following inclusion criteria were satisfied: documented hospitalization related to acute heart failure, and presence at admission of hyponatremia, identified by a serum sodium level of <135 mEq/L [9,10]. Clinical, echocardiographic and hematochemical features were noted for each enrolled patient, so as to collect sufficient data to build a Cox regression model providing multiple exposure variables. The outcome variables taken into account for the retrospective analysis were mortality from all causes and rehospitalizations, with a follow-up of 30 days after hospital discharge. The cohort of patients with ADHF was then divided into two subsets: the first group consisting of patients who came to hospital discharge with clinical picture improved with respect to admission, but characterized by a condition of persistent hyponatremia; and a second group composed of patients who, besides the improvement of clinical picture, had their serum sodium level normalized during the hospital stay until the time of discharge. A comparison was established between the two groups as regards the aforementioned endpoints of interest.

### Statistical Analysis

The two groups of patients, respectively with or without serum sodium normalized at discharge, were compared with regard to endpoints of interest (mortality from all causes and unplanned hospitalizations at a follow-up of 30 days after discharge). Continuous variables were expressed as mean ± standard deviation and were tested for normality of distribution using the Kolmogorov-Smirnov test. They were compared using one-way analysis of variance (ANOVA) and/or independent samples t-test for normally distributed continuous variables, or using Mann-Whitney U test for non-normally distributed continuous variables. Categorical variables were described as counts and percentages and compared using the chi-square test. Multivariable Cox proportional-hazards regression analysis for 30-day unplanned readmission or death was used to ascertain whether persistent hyponatremia at discharge was an independent predictor of 30-day unplanned readmission or death. Several variables were inserted into the model in addition to “persistent hyponatremia at discharge”. The variables used in this model were those that achieved statistical significance at *p* < 0.05 upon univariate analysis or those known to be a post-discharge mortality predictor based on prior studies. All statistical tests were performed with a commercially available statistical analysis program (SPSS 15.0 for Windows, SPSS Inc., Chicago, IL, USA). Statistical significance was assessed using a two-sided *p*-value. A *p*-value less than 0.05 was considered statistically significant.

## 3. Results

During the observational period, i.e., between April 2013 and April 2016, 842 patients in total were hospitalized with an admission diagnosis of ADHF in the two hospitals included in our retrospective study. Among these patients, 160 (19%) had a condition of hyponatremia, i.e., a serum sodium level of <135 mEq/L at admission.

**Figure 1:**
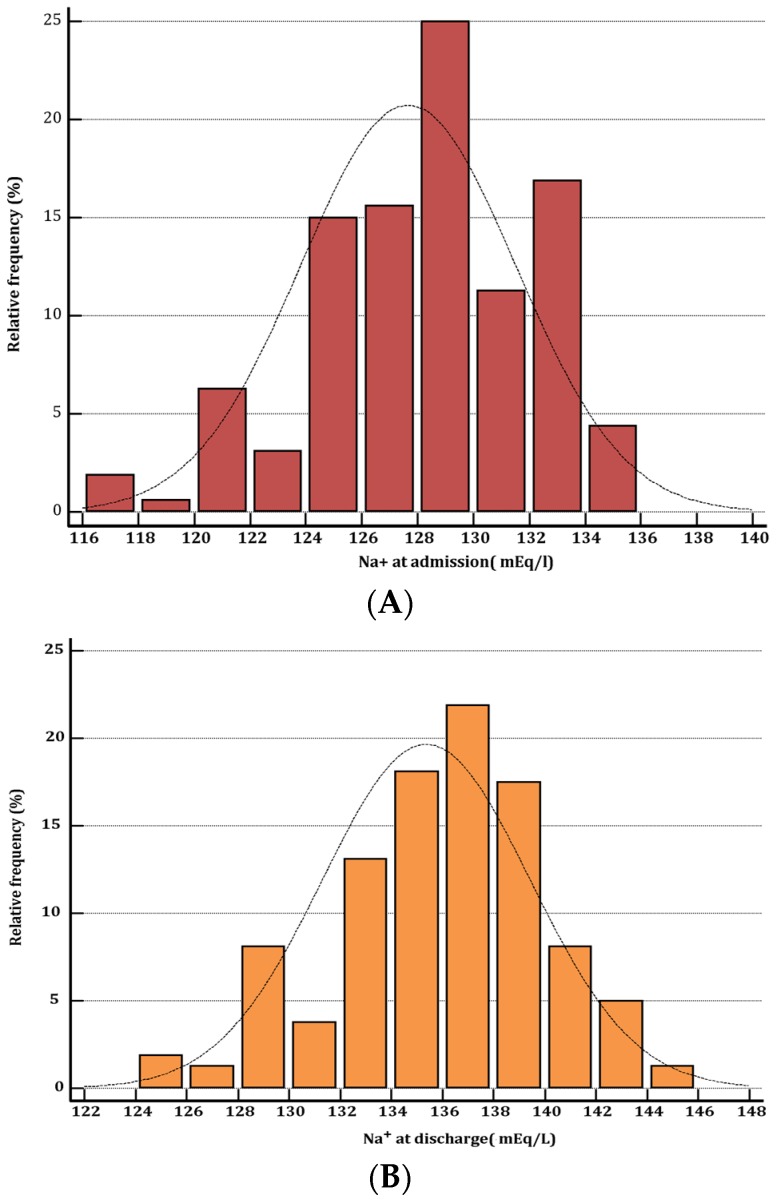
Distribution of serum sodium (Na^+^) in the population of 160 hyponatremic patients enrolled in our retrospective study. The distribution of serum Na^+^ values among the patients on admission is represented in histogram (**A**), while that of serum Na^+^ at the time of discharge is shown in histogram (**B**). The classes used for subdividing the values of serum Na^+^ correspond to intervals of 2 mEq/L.

**Figure 2:**
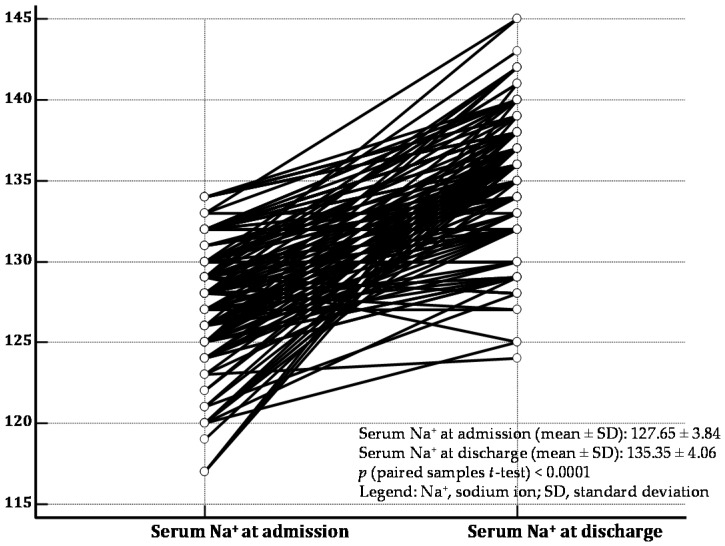
The changes of serum Na^+^ sodium between hospital admission and discharge are represented by means of a dot-and line diagram for each of the 160 patients with recent acute decompensated heart failure enrolled in the study. A significant increase in serum Na^+^ level is noticeable: *p* (paired samples *t*-test ) < 0.0001 . Nevertheless, 56 patients did not attain the cut-off of 135 mEq/L at discharge, so as to be regarded as affected by persistent hyponatremia.

Among the 160 ADHF patients affected by hyponatremia at hospital admission, persistent hyponatremia at hospital discharge was observed in 56 patients (35%). Baseline clinical, echocardiographic and hematochemical characteristics of patients are summarized in Table 1. Moreover, in Figure 1, the distribution of serum sodium values measured at hospital admission (Figure 1A) as well as at the time of hospital discharge (Figure 1B) is depicted. In addition, in Figure 2, the changes in serum sodium between admission and discharge for each patient are represented.

The study population was preliminarily subdivided into two groups, the former with persistent hyponatremia at discharge (56 patients) and the latter with serum sodium which increased during the hospital stay so as to attain the normal range at discharge (104 patients). The two groups did not show statistically significant differences as regards the values of serum Na^+^ measured at admission (130.5 (SD, 4.4) in the former versus 130.7 (SD, 5.5) mEq/L in the latter; *p* = 0.8148).

In the entire cohort of patients (including both the group with persistent hyponatremia and the one with serum Na^+^ normalized at discharge), 52 patients (32.5%) were affected by the composite endpoint “death or rehospitalization during a 30-day follow-up”.

Furthermore, the odds of having a 30-day unplanned readmission or death were significantly higher in patients with persistent hyponatremia compared to those who exhibited a serum Na^+^ normalized at discharge (crude odds ratio (OR) = 28.2; 95% CI: 11.58–68.62; *p* (chi-square) < 0.0001; see Table 2, Section I). In particular, the risk of death over a 30-day follow-up was not significantly different when comparing the group characterized by persistent hyponatremia with the one whose serum sodium became normal at discharge; on the contrary, the frequency of hospital readmissions was significantly higher in the former compared to the latter (see Table 2, Sections IIA and IIB).

Indeed, in the comparison between patients with versus those without persistent hyponatremia, the crude OR for “death within 30 days after discharge” was 1.256 (95% CI: 0.334–4.65; *p* (Fisher exact test) = 0.741) whereas, by comparing the two groups again using “hospital readmission within 30 days after discharge” as the outcome variable, the crude OR was 29.4 (95% CI: 10.93–79.05; *p* (chi-square) < 0.0001; see Table 2, Sections IIA and IIB).

In a Cox proportional-hazards multivariable regression model, persistent hyponatremia was associated with the increased probability of “death or readmission within 30 days after discharge” (adjusted hazard ratio = 3.0743; 95% CI: 1.3981–6.7601; *p* = 0.0054; see Table 3). The other exposure variables associated with the increased risk of the above-mentioned composite endpoint were “hospital admissions in the last 12 months” (*p* < 0.0001), “length of stay of index admission, days” (*p* = 0.0015) and “NYHA class III at discharge” (*p* = 0.0022; Table 3).

## 4. Discussion

Among the 160 patients with ADHF and hyponatremia at hospital admission enrolled in our retrospective study, 56 (35%) had hyponatremia that persisted at discharge. In these patients with persistent hyponatremia, a significantly higher odds of having an unplanned rehospitalization or death over a 30-day follow-up was found compared to patients with recent ADHF whose serum sodium had become normal during hospitalization until discharge (*p* = 0.0054 at multivariable Cox regression analysis). Moreover, based on the multivariate Cox regression analysis, the association between persistent hyponatremia and increased hazard of 30-day unplanned readmission or death seemed to be independent of heart failure severity.

Irrespective of any possible achievement of a normal serum sodium concentration, the Everest studies [11,12] demonstrated that in patients with cardiac decompensation, the use of tolvaptan, a vasopressin receptor antagonist, started during the hospital stay and then continued in the follow-up, was not associated with increased survival, but only with a significant increase in urine output and the consequent reduction of signs and symptoms of congestion, thereby improving the patient’s quality of life [11,12].

In addition, several previous studies [1,2,3,4,5] documented that persistent hyponatremia at discharge predicts increased mortality and hospital readmissions in patients hospitalized for ADHF. However, in these studies a comparison was made between ADHF patients with persistent hyponatremia at hospital discharge and patients with ADHF who had normal serum sodium levels at admission. Therefore, these studies are different from our research because they do not address the problem of verifying whether the return to normality of serum sodium in patients with cardiac decompensation and hyponatremia at baseline, i.e., at hospital admission, leads to a reduction of mortality and rehospitalizations with respect to patients who do not have their serum sodium normalized at discharge. Besides this, we have identified three studies [6,7,13] that, similarly to our research, assessed the impact of the normalization of serum sodium during hospitalization on mortality and readmissions: two are post-hoc analyses of randomized controlled trials, which provide conflicting results [6,7], and one is a retrospective cohort study, which seems to show a favorable influence of hyponatremia correction on the short-term risk of death and hospitalizations, compared with the absence of correction of hyponatremia over the course of hospitalization [13].

In particular, in the Acute and Chronic Therapeutic Impact of a Vasopressin Antagonist in Chronic Heart Failure (ACTIV in CHF) trial, Rossi et al. [6] detected a reduced 60-day mortality in patients who increased their serum Na^+^ by ≥2 mEq/L between admission and the last Na^+^ level available in an every-other-week post-hospital discharge follow-up. Conversely, in the Outcomes of a Prospective Trial of Intravenous Milrinone for Exacerbations of Chronic Heart Failure (OPTIME-CHF) study, Klein et al. [7] did not detect different mortality among 244 patients who had an admission Na^+^ level in the lowest quartile.

Additionally, using a multivariable logistic regression model, the study by Donzè et al. [13] ascertained that the absence of correction of hyponatremia over the course of hospitalization was independently associated with a significant 45% increase in the odds of having a 30-day unscheduled rehospitalization or death (composite endpoint). Moreover, in this study, logistic regression analyses, separately performed for each of the two components of the primary endpoint, demonstrated that both 30-day mortality and unscheduled hospital readmissions were significantly reduced in patients who had their serum sodium normalized at discharge.

Our study is in agreement with the study of Donzè et al. [13] as regards the favorable impact of hyponatremia correction on the primary composite endpoint (30-day unscheduled readmissions or death), but it does not show a significant improvement in the secondary endpoint “30-day mortality”.

The mechanisms by which persistent hyponatremia is associated with an increased risk for 30-day unscheduled readmissions and death are still a matter of debate.

In accordance with other authors [14], our data do not support the hypothesis that hyponatremia depends on the severity of heart failure. Indeed, based on the Cox regression analysis, in our study, persistent hyponatremia appears to be an independent predictor of 30-day unplanned rehospitalization or death. Moreover, the thesis that confounding factors such as heart failure severity would be responsible for the association found between persistent hyponatremia at discharge and adverse events during follow-up seems to be unlikely, because previous data have shown that sodium levels do not differ significantly according to the ejection fraction [14]. By contrast, we suppose that persistent hyponatremia may be a marker of comorbidities which in turn increase the risk of readmissions. 

### Study Limitations

We did not evaluate the medical therapy during the hospital stay. Therefore, a lack of adequate medical therapy may have been responsible for the persistence of hyponatremia in some or all of the patients. However, retrospective assessment of the therapy administered for correction of hyponatremia during hospitalization in patients with heart failure, with a detailed list of the adopted therapeutic measures, scrupulous recording of doses and comparative evaluation of the outcomes of different treatment regimens, may be the subject of a future study of our research team.

## 5. Conclusions

In the present retrospective study, the risk of 30-day rehospitalization or death was significantly higher in ADHF patients with persistent hyponatremia in comparison with those who had had their serum sodium normalized during the hospital stay. This association seemed not to ensue from possible differences between the two groups as regards heart failure severity.

## Figures and Tables

**Table 1:** Baseline characteristics.

Variable	Persistent Hyponatremia at Discharge (No. 56 pts)	Serum Sodium Normalized at Discharge (No. 104 pts)	*p*-Value
Age, year, mean (SD)	76 (10.4)	77 (10.8)	0.5723
Male gender, No. (%)	28 (50)	54 (51.9)	0.9471
Previous hypertension, No. (%)	30 (53.5)	52 (50)	0.7908
Coronary artery disease, No. (%)	24 (42.2)	49 (46.2)	0.7268
COPD, No. (%)	10 (17.85)	19 (18.26)	0.8803
Diabetes, No. (%)	16 (28.5)	35 (33.65)	0.6311
Chronic kidney disease, (eGFR < 60 mL/min/1.73 m^2^) No. (%)	23 (41)	41 (39.4)	0.9730
Atrial flutter/ fibrillation, No. (%)	17 (30.3)	28 (0.27)	0.7822
LVEF (%), mean (SD)	40 (6.6)	40 (7.5)	1.00
SBP, mm Hg, mean (SD)	105 (20)	112 (21)	0.043 *
Heart rate, bts/ min at admission, mean (SD)	99 (15)	95 (15)	0.1096
HFREF, No. (%)	29 (51.7)	51 (49)	0.8684
HFPEF, No. (%)	27 (48.3)	53 (51)	0.8684
Hb (g/dl), mean (SD)	11.5 (0.6)	11.3 (0.8)	0.1034
serum Na^+^, mEq/L at admission, mean (SD)	130.5 (4.4)	130.7 (5.5)	0.8148
serum creatinine, mg/dL, mean (SD)	1.3 (0.2)	1.25 (0.3)	0.2646
eGFR, mL/ min (admission), mean (SD)	60 (12)	64 (9)	0.0186 *
BNP, pg/mL (admission), mean (SD)	1100 (120)	1058 (160)	0.0874
fluid removal after 48 h, L	5.5 (1.8)	5.050 (2.4)	0.2210
no. of admissions in the last 12 months, median(IQR)	2.0 (0–3)	0.5 (0–1)	0.0182 *^,‡^
length of stay of index admission, days, median (IQR)	7.0 (4.75–8)	5.0 (4–5)	0.0186 *^,‡^

Abbreviations: y, years; SD, standard deviation; COPD, chronic obstructive pulmonary disease; eGFR, estimated glomerular filtration rate; LVEF, left ventricular ejection fraction; SBP, systolic blood pressure; HFREF, heart failure with reduced left ventricular ejection fraction; HFPEF, heart failure with preserved left ventricular ejection fraction; Hb, hemoglobin; BNP, B-type natriuretic peptide; IQR, interquartile range. * *p* < 0.05 ; ^‡^ Mann-Whitney test (independent samples).

**Table 2:** (I) In the 2 × 2 contingency table, the probability of death or rehospitalization evaluated in 160 patients with ADHF characterized by hyponatremia at admission is represented. Based on the reported data, the odds of having a 30-day unplanned readmission or death was much higher in patients with persistent hyponatremia compared to those who exhibited a sodium level normalized at discharge (odds ratio = 28.2; 95% CI: 11.58 to 68.62); (IIA) The odds of all-cause mortality within 30 days from discharge, detected in the group of ADHF patients with hyponatremia at admission which was not corrected during hospital stay (persistent hyponatremia: yes) and in the group of ADHF patients with initial hyponatremia and normalized serum sodium at discharge (persistent hyponatremia: no) are compared. The comparison shows that the normalization of serum sodium during the hospital stay was not associated with the decreased probability of short-term exitus in comparison with patients with persistent hyponatremia; (IIB) The odds of short-term re-hospitalization were significantly higher in patients with persistent hyponatremia in comparison with those who had had their serum sodium normalized (OR = 29.4; 95% CI: 10.93 to 79.05; *p* < 0.0001). ADHF, acute decompensated heart failure; OR, odds ratio; CI, confidence interval.

**I**		**Death or Rehospitalization during 30-Day Follow-Up**		
		**Yes**	**No**	**Total**
**Persistent hyponatremia**	**Yes**	42	14	56
	**No**	10	94	104
	**Total**	52	108	160
**Single table analysis:**				
**Odds ratio**		**95% Confidence Interval**		***p*-value**
28.2		11.58–68.62		*p* < 0.0001
**IIA**		**Death within 30 Days after Discharge**		
		**Yes**	**No**	**Total**
**Persistent hyponatremia**	**Yes**	4	52	56
	**No**	6	98	104
	**Total**	10	150	160
**Single table analysis:**				
**Odds Ratio**		**95% Confidence Interval**		***p* (Fisher Exact Test)**
1.256		0.334–4.65		0.7410
**IIB**		**Readmission within 30 Days after Discharge**		
		**Yes**	**No**	**Total**
**Persistent hyponatremia**	**Yes**	36	20	56
	**No**	6	98	104
	**Total**	42	118	160
**Single table analysis:**				
**Odds Ratio**		**95% Confidence Interval**	***p* (Chi Square Corrected According to Yates)**	
29.4		10.93–79.05	<0.0001	

**Table 3:** Multivariable Cox proportional-hazards regression for 30-day unplanned readmission or death.

Variable	Hazard Ratio	95% CI	*p*-Value
Persistent hyponatremia	3.0743	1.3981–6.7601	0.0054
Age	1.0018	0.9668–1.0380	0.9235
Male sex	0.9982	0.5486–1.7223	0.5038
Hospital admissions in the last 12 months	2.0004	1.5171–2.6378	<0.0001
Length of stay of index admission	1.4951	1.1680–1.9138	0.0015
Coronary artery disease	1.2155	0.6003–2.4612	0.5896
COPD	1.4590	0.7663–2.7779	0.2526
Diabetes	0.8538	0.4145–1.7588	0.6698
Chronic kidney disease	0.8053	0.4289–1.5122	0.5028
Atrial flutter/fibrillation	1.0400	0.4881–2.2160	0.9195
NYHA class III at discharge	3.0125	1.4942–6.0736	0.0022

CI, confidence interval; COPD, chronic obstructive pulmonary disease.
